# Supplementary material for: Identification of photoperiod-induced specific miRNAs in the adrenal glands of Sunite sheep (Ovis aries)
Source: Front Vet Sci. 2022 Jul 22;9:888207. doi: 10.3389/fvets.2022.888207 (PMC9354845; doi:10.3389/fvets.2022.888207)
Supplement: Supplementary file 1 [file Data_Sheet_1.ZIP › Supplementary file/miRDeep2 file.zip!/miRDeep2 file/Know_Structure/LP42A1/oar-let-7c.pdf]

miRBase precursor : oar-let-7c
Total read count : 126280
oar-let-7c read count : 126201
remaining reads : 79

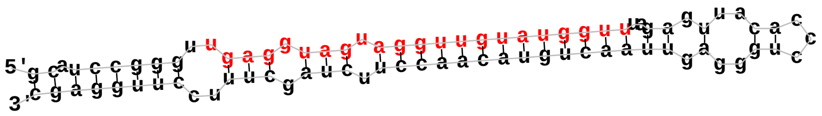

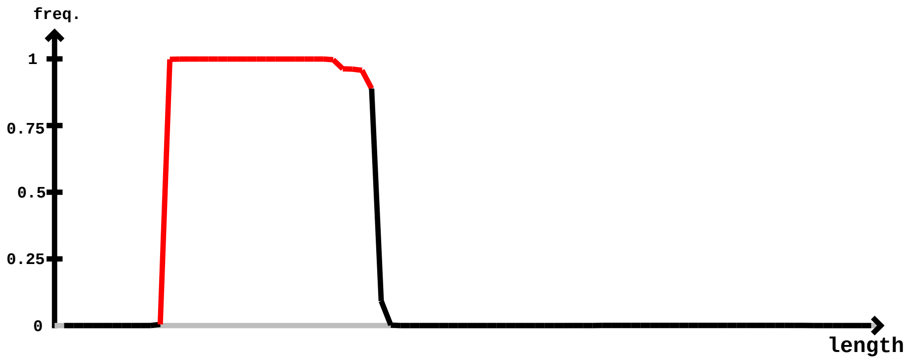

oar-let-7c

```
5'- gcauccgggu ugagguaguagguuguaugguu uagaguuacacccugggaguuaacuguacaaccuucuagcuuuccuuggagc   -3'    exp
    ((.((((((..(((.(((.(((((((((((((..((.(..((...))..).))))))))))))))).))).)))..))))))))   reads       mm          sample
    ........guugagguaguagguuguaugg......................................................   2           0           seq
    ........guugagguaguagguuguauggu.....................................................   5           0           seq
    ........guugagguaguagguuguauAguu....................................................   2           1           seq
    .........uugagguaguagguuguau........................................................   2           0           seq
    .........uugagguaguagguuguaug.......................................................   1           0           seq
    .........uugagguaguagguuguauA.......................................................   1           1           seq
    .........uugagguaguagguuguaugg......................................................   10          0           seq
    .........uugagguaguagguuguauAgu.....................................................   4           1           seq
    .........uugagguaguagguuguauggu.....................................................   158         0           seq
    .........uugagguaguagguuguaugguu....................................................   330         0           seq
    .........uugagguaguagguuguauAguu....................................................   10          1           seq
    .........uugagguaguagguuguaugguuu...................................................   25          0           seq
    ..........ugagguaguUgguugua.........................................................   2           1           seq
    ..........ugagguagGagguugua.........................................................   31          1           seq
    ..........ugagguaguaAguugua.........................................................   7           1           seq
    ..........ugagguaguagguugua.........................................................   277         0           seq
    ..........ugaggCaguagguugua.........................................................   1           1           seq
    ..........ugagguaguagguuUuau........................................................   6           1           seq
    ..........ugagguaguagguuguau........................................................   3846        0           seq
    ..........ugaAguaguagguuguau........................................................   44          1           seq
    ..........ugagguUguagguuguau........................................................   2           1           seq
    ..........ugagguaguaAguuguau........................................................   25          1           seq
    ..........ugagguagGagguuguau........................................................   381         1           seq
    ..........ugagguaguagguuguaug.......................................................   150         0           seq
    ..........ugagguaguagguuguaugg......................................................   538         0           seq
    ..........ugagguaguagguuguauggu.....................................................   8477        0           seq
    ..........ugagguaguagguuguaugguu....................................................   100097      0           seq
    ..........ugagguaguagguuguaugguuu...................................................   11433       0           seq
    ..........ugagguaguagguuguauAguuua..................................................   121         1           seq
    ..........ugagguaguagguuguaugguuua..................................................   73          0           seq
    ..........ugagguaguagguuguaugguuuag.................................................   1           0           seq
    ..........ugagguaguagguuguauAguuuag.................................................   1           1           seq
    ..........ugagguaguagguuguaugguuuagagu..............................................   1           0           seq
    ...........gagguaguagguuguau........................................................   1           0           seq
    ...........gagguaguagguuguauggu.....................................................   10          0           seq
```

oar-let-7c

```
gcauccggguugagguaguagguuguaugguuuagaguuacacccugggaguuaacuguacaaccuucuagcuuuccuuggagc
...........gagguaguagguuguaugguu....................................................   111   0   seq
...........gagguaguagguuguaugguuu...................................................   9     0   seq
............agguaguagguuguaugguu....................................................   1     0   seq
............agguaguagguuguaugguuu...................................................   3     0   seq
.............gguaguagguuguaugguu....................................................   2     0   seq
...............uaguagguuguauAguuuaga................................................   1     1   seq
................................uagaguuacacccugggaguua..............................   2     0   seq
........................................cacccugggaguuaacuguacaacc...................   1     0   seq
.................................................aguuaacuguacaaccuucuagcu...........   2     0   seq
...................................................uuaacuguacaaccuucuagcuu..........   1     0   seq
....................................................uaacuguacaaccuucuagcuu..........   2     0   seq
.......................................................cuguacaaccuucuagcuuu.........   4     0   seq
.......................................................cuguacaaccuucuagcuuuc........   6     0   seq
.......................................................cuguacaaccuucuagcuuucc.......   47    0   seq
.......................................................cuguacaaccuucuagcuuuccu......   10    0   seq
........................................................uguacaaccuucuagcuuuccu......   3     0   seq
```
